# Tetramethylenedisulfotetramine: A Health Risk Compound and a Potential Chemical Warfare Agent

**DOI:** 10.3390/toxics6030051

**Published:** 2018-08-22

**Authors:** Jiří Patocka, Tanos C. C. Franca, Qinghua Wu, Kamil Kuca

**Affiliations:** 1 Department of Radiology and Toxicology, Faculty of Health and Social Studies, University of South Bohemia Ceske Budejovice, 370 05 Ceske Budejovice, Czech Republic; toxicology@toxicology.cz; 2 Biomedical Research Center, University Hospital Hradec Kralove, 500 03 Hradec Kralove, Czech Republic; 3 Laboratory of Molecular Modeling Applied to the Chemical and Biological Defense (LMCBD), Military Institute of Engineering, Rio de Janeiro 22290-270, RJ, Brazil; tanos@ime.eb.br; 4 Center for Basic and Applied Research, Faculty of Informatics and Management, University of Hradec Kralove, 500 03 Hradec Kralove, Czech Republic; 5 College of Life Science, Yangtze University, Jingzhou 434025, China; 6 Department of Chemistry, Faculty of Science, University of Hradec Kralove, 500 03 Hradec Kralove, Czech Republic; 7 Department of Cellular Biology and Pharmacology, College of Medicine, Florida International University, Miami, FL 33199, USA

**Keywords:** Tetramethylenedisulfotetramine, TETS, pesticide, rodenticide, chemical warfare agent

## Abstract

Tetramethylenedisulfotetramine (TETS, tetramine) is a toxic organic compound that is used as an effective rodenticide. However, this neurotoxin is not only toxic to rodents, it also causes poisoning in humans. Due to its high level of toxicity for humans, the use of TETS as a rodenticide has been banned and its production has been discontinued. Despite this, human poisoning by this substance is unfortunately still very common. The largest number of poisonings are reported in China, but in the United States, dozens of poisonings still happen annually. TETS is one of the most hazardous pesticides and also a possible chemical warfare agent with no known antidote. In this article, we aim to summarize the biochemical and toxicological data of TETS and hope to cast some light on the toxicological risk to human health.

## 1. Introduction

Tetramethylenedisulfotetramine (TETS) is a highly toxic convulsant and a potent antagonist of γ-aminobutyric acid (GABA), leading to excitation. This organic compound was developed as an effective rodenticide, but the high toxicity to mammals led to it being banned in most countries [1]. The production and use of TETS has been banned worldwide since 1984, however due to the continuing demand and its ease of production, this chemical is still readily available in China as rat poison [2]. In China, TETS is available on a rural black market under the name “Du Shu Qiang” [3]. TETS is 100 times more toxic than potassium cyanide; with a lethal dose for humans of 7–10 mg and no known antidote [4]. Acute intoxication with TETS can cause vomiting, convulsions, status epilepticus and even death. Individuals who survive poisoning may exhibit long-term neuropsychological issues and cognitive deficits [5]. TETS has been included in the World Health Organization’s (WHO) list of “extremely hazardous” pesticides [6]. In China, over 14,000 cases of TETS intoxication occurred between 1991 and 2010, and 932 of these lead to death [6]. However, the age and gender differences have not been analysed. Moreover, numerous poisonings have occurred worldwide, including Europe and the United States. In 2002, the first known case of human illness in the United States caused by TETS occurred in New York City [4]. Most cases of intoxication by TETS are due to accidental ingestion and the most recognized complication is status epilepticus. The poisoning can be fatal within hours [4]. To date, there is no standardized therapy for TETS poisoning, but pyridoxine and chelation therapy seem to be more effective [4,5]. It is noteworthy that TETS is considered a chemical threat agent, since it has the capacity to cause mass casualties if released accidentally or as an act of terrorism [7,8].

Following a recent case report confirming the presence of TETS in the United States, and taking into consideration the toxicity of this compound, the absence of an antidote, and the potential of its utilization as an agent of intentional mass poisoning, educational efforts have been undertaken in many countries to inform the public about this potentially lethal rodenticide [9]. In this short review, we have summarized the chemical, biochemical, environmental and toxicological data currently available in the literature about TETS, with the goal of providing the scientific community with useful information that could be helpful in the discovery of new means of preventing intoxication and/or developing antidotes against TETS.

## 2. Chemistry

**Figure 1:**
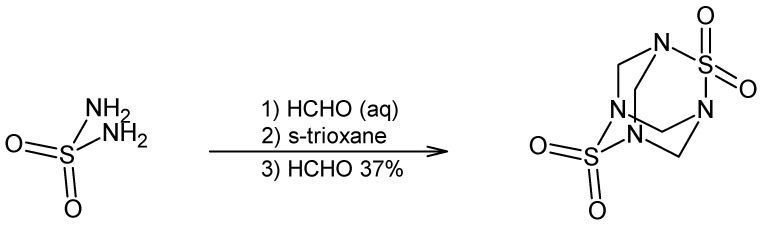
Synthesis of TETS.

TETS (2,6-dithia-1,3,5,7-tetraazatricyclo[3.3.1.13,7]-decane-2,2,6,6-tetraoxide, CAS Registry No. 80-12-6) is an odourless, tasteless, white crystalline powder with a melting point between 255 °C and 260 °C, slightly soluble in water, dimethylsulfoxide and acetone; insoluble in methanol and ethanol, with Log P (octanol−water) = −3.520 [1,4,10]. This highly toxic heteroadamantane rodenticide was first synthesized in 1933 through the condensation of sulfamide and formaldehyde (Figure 1) and is usually used in pillows and upholstery as an impregnating stiffening and anti-mould agent [1,10].

## 3. Toxicokinetics and Toxicodynamics

TETS is absorbed rapidly through the gastrointestinal tract. When a person drinks TETS-contaminated milk or water, the acute toxicity can normally be observed within 1–5 min [11]. The time from ingestion to the display of observed symptoms ranges from several minutes to half an hour. TETS is slowly eliminated from the body. In a report, urinary TETS can be detected in 60% of cases after 10 days post-ingestion [12]. In a patient with acute TETS poisoning, the urinary clearance of TETS was 60 µg/24 h. A total of 80 µg of this poison was excreted via the urine within 48 h [13]. To date, there is no data available on the distribution and biological half-life of TETS, while the available toxicokinetic data is incomplete and sometimes contradictory.

The clinical manifestations of the TETS toxicity are seizures [14]. The chloride channels on the γ-aminobutyric acid (GABA) receptor are the major binding hub for TETS. Normally, this binding is selective and irreversible. Very recently, α_2_β_3_γ_2_ was shown to be the most important GABA_A_ receptor for the seizure-inducing activity of TETS [15]. The regulation of chloride in the neuron is therefore disrupted [16]. The toxicant mechanism of TETS is not fully known but it is assumed that DNA damage in the cells could be related to the action of this compound [17]. As shown by Wang et al., [18] Bcl-2 and caspase-3 are involved in the pathophysiological action of TETS in poisoned rats and the target organs are the heart, brain and liver [19]. TETS also dramatically affects Ca^2+^ dynamics in cultured hippocampal neurons and it causes an immediate but transient elevation of neuronal intracellular Ca^2+^. The effect of TETS on Ca^2+^ dynamics requires the activation of N-methyl-D-aspartic acid (NMDA) receptors [20]. Combined treatment with diazepam and allopregnanolone reverses TETS-induced Ca^2+^ dysregulation in cultured neurons and protects TETS-intoxicated mice against lethal seizures [21].

## 4. Toxicity

In most spices, the acute toxicity of TETS, in LD_50_ values, is 0.1–0.3 mg/kg of body weight (Table 1). Rabbits dosed with TETS (0.4 mg/kg body weight) and killed after 1 h showed detectable levels (0.07 to 0.238 mg/g) of TETS in the liver [22]. The CD_50_ values of TETS for clonic and tonic seizures in mice were 0.11 and 0.22 mg/kg, respectively [7]. The brain’s inflammatory response can also be observed in the toxicity of TETS [7]. The neurobehavioral consequences of TETS exposure, described in human survivors of acute TETS intoxication, are due to sustained seizure activity rather than a direct effect of the chemical itself [5]. Realistic models of exposure, behavioural assessments and multifaceted treatment investigations are needed to elaborate the toxicity of TETS [23]. Very recently, Lauková et al. [24] implemented an investigation in developing rats of both sexes to identify any potential age- and sex-dependent vulnerability to TETS exposure. The authors showed that the youngest rats represented the most vulnerable population to the TETS-induced toxidrome. Females appeared to be more vulnerable than males. TETS exposure promotes seizure spread and progression in survivors. Currently, there is no such data on humans and this study can cast some light on the human clinical study of TETS poisonings. 

Mild symptoms of TETS poisoning include headaches, dizziness and fatigue. Severe clinical features include foaming, consciousness reduction, seizures, urinary incontinence, coma and death from respiratory failure [12,25]. Li et al. [6] reported a series of nine people with TETS intoxication presenting convulsive status epilepticus (CSE) as the initial manifestation. The median duration of the CSE after admission was 6 h. All had normal neuro-imaging, but an interictal EEG showed bilateral epileptic waves. Multiple organ dysfunction syndrome occurred in six people, of whom three died. 

TETS is stable in tissues and can therefore cause secondary poisoning. Due to the stability of this pesticide in tissues, birds and scavenging animals can be poisoned. In a similar manner, humans may also be poisoned [26,27]. The seizures caused due to the blocking of the GABA receptors can persist for years after the initial poisoning. Sodium valproate is a good choice for the treatment of TETS-induced epilepsy [28].

In forensic practice, TETS poisoning should be considered when the patient has signs of abnormal excitation of the CNS, hyperspasmia, cerebral haemorrhage and convulsions [29]. Tissues and urine can be used to test for TETS [22].

## 5. Routes of Human Exposure

The ingestion of contaminated food is the most commonly reported route for human exposure to TETS, including cases of suicide and homicide [12,29,33]. Occupational poisoning via the respiratory tract is also commonly reported [29], while two cases of acute TETS poisoning were reported due to dermal exposure [29].

The lethal dose of TETS in humans is similar to that in rodents (0.1 mg/kg) [34]. The total lethal dose for a human is approximately 10 mg with a reported fatality rate as high as 3.67% [35,36]. The toxicity of TETS in children is similar to adults. TETS is excreted in the urine and this can be used for further forensic investigations [37].

## 6. Biomedical Investigations

### 6.1. Biochemistry Examination

Thus far, there is no specific biomedical marker that would allow TETS poisoning to be distinguished from poisoning by other pesticides. All the detected changes are nonspecific. Increased concentrations of bilirubin, aspartate aminotransferase (AST), lactate dehydrogenase (LDH) and creatine kinase (CK) in the blood from patients with severe acute TETS poisoning are reported. These values are correlated with the severity of the poisoning [12,38,39]. The CK increase is a result of muscular contraction during convulsions [39]. Proteinuria and hematuria have been reported [38]. The white cells are increased in most acute TETS poisoning patients [12].

### 6.2. Electrocardiogram (ECG) Examination

Tachycardia, bradycardia, premature beat and changes indicating toxic myocarditis. It also involves the flatness or inversion of the T wave, and the elevation or depression of the ST segment [38,39]. Normally, the ECG changes are reversible; however one patient with acute poisoning developed Adam–Stokes syndrome and continued to show T wave changes after three years [12].

### 6.3. Electroencephalogram (EEG) Examination

Paroxysmal sharp waves diffuse theta waves, and delta waves are observed as a result of poisoning by TETS. Most patients returned to a normal status after two weeks. The longest recovery was three months [38,39]. The CT results were normal in 15 detected clinical cases [40].

## 7. Analytical Investigations

A gas chromatography (GC) method for TETS determination has been conducted by Wu et al. [41], an average recovery of 89.8% and a detection limit of 1.00 ng was achieved for TETS. For the detection of TETS in food and tissues, a GC-nitrogen-phosphorus detector (NPD) method was performed and reached a limit of detection of 0.02 ng [42]. Zeng et al. [43] also developed an analytical method for determining TETS in human urine by GC-flame thermionic detection (GC-FTD) coupled with direct immersed solid-phase micro-extraction (DI-SPME). In their method, the limit of the quantitation of TETS in urine was 0.082 ng/mL. Owens et al. [44] further developed an LC-MS method for the quantitation of TETS spiked in beverages including milk, juice, tea, cola and water. Quantitation by LC-MS/MS was based upon m/z 347 to 268. The limit of quantitation was 0.10 μg/mL. The solid phase microextraction of TETS coupled with GC NPD was also performed by the authors in [45,46]. The analysis of TETS in a series of biological samples by GC-MS has been described [47,48]. The typical peaks are m/z 240, 212, 185, 132, 121, 92, 76 and 42 [47]. GC-MS is sensitive but not rapid or high-throughput. Very recently, Vasylieva et al. [49] developed an immunoassay specific to TETS with an IC_50_ of 4.5 ± 1.2 ng/mL and a limit of detection of 0.2 ng/mL.

## 8. Treatments

Currently, a standardized therapy for TETS poisoning has not been established [37]. However, with the clinical treatment of large numbers of patients with TETS poisoning, some clinical therapies seem to be efficient for this toxicosis. In 2001, Li and Zeng clinically analysed 15 cases involving TETS in China [50]. Gastric lavage in the early stage shows a good effect on this toxicosis. Patients (13 cases) with convulsions got immediate relief after plasma exchange. The use of a large dosage of tranquillizer resulted in a better control of convulsion in four cases treated with a ventilator than in the others. Plasma exchange, tracheotomy and mechanical ventilation are the most effective treatment methods [37]. Pretreatment with sodium bromide or long-acting barbiturates increased the latency and reduced the severity of TMDT-induced seizures. Repeated benzodiazepine dosing or the combined application of benzodiazepines and NMDA receptor antagonists are more likely to be effective in treating TETS poisoning [3]. Moreover, a sequential combination treatment with benzodiazepine diazepam followed by the NMDA receptor antagonist dizocilpine (MK-801) is more effective than either individual therapy or simultaneous administration of the two agents in treating TETS poisoning [8]. In addition, Several GABA receptor modulators, including midazolam, flurazepam, avermectin Ba1, baclofen, isoguvacine and propofol, are candidate antidotes for TETS [51].

## 9. Conclusions

TETS meets the criteria for inclusion in the list of extremely hazardous pesticides maintained by the WHO and is even more lethal than the WHO’s most toxic registered pesticide, sodium fluoroacetate. Pesticide and rodenticide poisonings are a serious threat to populations. Currently, the use of banned rodenticide (such as TETS), with its associated lethality, is a serious public health concern. The extreme toxicity of TETS, the absence of a specific antidote, the lack of properties that enable detection, and the possibility of using this compound in mass poisonings indicate that TETS can be considered a potential chemical agent. However, the absorption, distribution, metabolism and excretion of TETS are still not clear. In addition, the age and sex differences in susceptibility to TETS have not been fully explored. In the future, an analysis of TETS poisoning on the basis of gender, age, education, geographic area and type of work is warranted.

## Figures and Tables

**Table 1:** Some toxicity parameters of Tetramethylenedisulfotetramine for mammals via different routes of administration.

Organism	Test type	Route	Dose	Reference
Mammal (species unspecified)	LD_50_	oral	0.10 mg/kg	[30]
mouse	LD_50_	intraperitoneal	0.21 mg/kg	[31]
mouse	LD_Lo_	oral	0.20 mg/kg	[32]
mouse	LD_Lo_	subcutaneous	0.10 mg/kg	[32]
rabbits	LD_50_	oral	0.40 mg/kg	[22]
